# Functional Characterization of the Receiver Domain for Phosphorelay Control in Hybrid Sensor Kinases

**DOI:** 10.1371/journal.pone.0132598

**Published:** 2015-07-07

**Authors:** Emiko Kinoshita-Kikuta, Eiji Kinoshita, Yoko Eguchi, Shiho Yanagihara, Keisuke Edahiro, Yuki Inoue, Momoka Taniguchi, Myu Yoshida, Kaneyoshi Yamamoto, Hirotaka Takahashi, Tatsuya Sawasaki, Ryutaro Utsumi, Tohru Koike

**Affiliations:** 1 Department of Functional Molecular Science, Institute of Biomedical and Health Sciences, Hiroshima University, Hiroshima, Japan; 2 Department of Science and Technology on Food Safety, Faculty of Biology-Oriented Science and Technology, Kinki University, Kinokawa, Japan; 3 Department of Frontier Bioscience, Hosei University, Koganei, Japan; 4 Division of Cell-Free Sciences, Proteo-Science Center, Ehime University, Matsuyama, Japan; 5 Department of Bioscience, Graduate School of Agriculture, Kinki University, Nara Japan; Shanghai Jiao Tong University, CHINA

## Abstract

Hybrid sensor kinase, which contains a histidine kinase (HK) domain, a receiver domain, and a histidine-containing phosphotransmitter (HPt) domain, conveys signals to its cognate response regulator by means of a His-Asp-His-Asp phosphorelay. We examined the multistep phosphorelay of a recombinant EvgAS system in *Escherichia coli* and performed *in vitro* quantitative analyses of phosphorylation by using Phos-tag SDS-PAGE. Replacement of Asp in the receiver domain of EvgS by Ala markedly promoted phosphorylation at His in the HK domain compared with that in wild-type EvgS. Similar Ala-substituted mutants of other hybrid sensor kinases BarA and ArcB showed similar characteristics. In the presence of sufficient ATP, autophosphorylation of the HK domain in the mutant progressed efficiently with nearly pseudo-first-order kinetics until the phosphorylation ratio reached a plateau value of more than 95% within 60 min, and the value was maintained until 180 min. However, both wild-type EvgS and the Ala-substituted mutant of His in the HPt domain showed a phosphorylation ratio of less than 25%, which gradually decreased after 10 min. These results showed that the phosphorylation level is regulated negatively by the receiver domain. The receiver domain therefore plays a crucial role in controlling the phosphorelay to the response regulator. Furthermore, our *in vitro* assays confirmed the existence of a similar hyperphosphorylation reaction in the HK domain of the EvgS mutant in which the Asp residue was replaced with Ala, confirming the validity of the control mechanism proposed from profiling of phosphorylation *in vitro*.

## Introduction

Bacterial and plant cells have a two-component signal-transduction regulatory system for certain cellular responses [1, 2]. In general, this system consists of a histidine sensor kinase and a response regulator. The histidine sensor kinase monitors an environmental stimulus and modulates the function of its cognate response regulator through protein phosphorylation. Accordingly, the response regulator mediates certain changes in gene expression or cell behavior. A typical histidine sensor kinase has a histidine kinase (HK) domain containing an invariant His residue that is autophosphorylated in an ATP-dependent manner, whereas a typical response regulator has a receiver domain containing a conserved Asp residue that can acquire a phosphoryl group from its cognate histidine sensor kinase. Most two-component systems have this type of a simple His-Asp phosphorelay design. However, some histidine sensor kinases, known as hybrid sensor kinases, have a more complex type of phosphorelay consisting of two additional domains: a receiver domain containing a conserved Asp residue and a histidine-containing phosphotransmitter (HPt) domain. In such cases, signals are transmitted through a His-Asp-His-Asp phosphorelay: a phosphoryl group moves successively from the HK domain to the first receiver domain, the HPt domain, and finally the receiver domain of the response regulator.

*Escherichia coli* K-12 has at least four types of hybrid sensor kinase, EvgS, BarA, ArcB, and TorS [2]. Although several mutagenesis and biochemical studies have demonstrated that all of these sensor kinases convey signals to their cognate response regulators by means of the universal four-step His-Asp-His-Asp phosphorelay [3–9], the kinetics of the multistep phosphorelay remains unclear. One reason for this is that identification of site-specific phosphorylation of His or Asp is technically difficult because of the labile nature of the phosphorylated amino acid residues. Since the standard free energies for a phosphoimidazole P–N bond on His and an acyl-phosphate P–C bond on Asp are larger than that for a phosphomonoester P–O bond on Ser, Thr, or Tyr, the phosphorylated His and Asp have the potential to serve as more reactive intermediates in phosphotransfer reactions. Therefore, in addition to Ser-, Thr-, and Tyr-phosphorylated proteins, His- and Asp-phosphorylated proteins play crucial roles as a sensor apparatus and a response regulator, respectively, of the two-component system in quick response to intra- and extra-cellular signals in prokaryotes as well as in plants. The phosphoryl group on His is thus rapidly hydrolyzed under acidic conditions and it has a half-life of several days under neutral conditions [10]. The phosphoryl group on Asp is extremely labile under both acidic and alkaline conditions and it has a half-life of several hours under neutral conditions [1, 10]. Consequently, neither a phosphoproteomic strategy using mass spectrometry nor a Western blotting procedure using phosphorylation site-specific antibodies can be used in the analysis of these phosphoproteins.

In this study, we therefore used Phos-tag SDS-PAGE, a technique that is capable of separating multiple phosphoprotein species that contain identical numbers of phosphoryl groups, but in which the phosphoryl groups are attached at different locations within the protein molecules [10–14]. The Phos-tag SDS-PAGE technique offers the following significant advantages: i) the phosphate-affinity procedure is almost identical to that for conventional SDS-PAGE; ii) a downstream procedure, such as gel staining, Western blotting, or mass spectrometric analysis, can be applied; iii) radioactive and chemical labels are unnecessary for kinase and phosphatase assays; iv) various phosphoprotein species, depending on the phosphorylation status, can be detected separately as multiple migration bands; v) the phosphate-binding specificity is independent of the kind of phosphorylated amino acid; vi) several phosphoprotein species having the same number of phosphate groups could be separated; vii) the time-course quantitative ratio of phosphorylated to nonphosphorylated proteins can be determined; and viii) unstable His- and Asp-phosphorylated proteins involved in a two-component signaling system can be detected simultaneously during their phosphotransfer reactions. Thus, this permitted the qualitative and quantitative detection of three types of phosphorylated species derived from the HK domain, the receiver domain, and the HPt domain, respectively, in three types of hybrid sensor kinases, EvgS, BarA, and ArcB, as produced by kinase reactions. This, in turn, allowed us to elucidate details of the kinetics of His- and Asp-phosphorylation in hybrid sensor kinases.

## Materials and Methods

### Materials

Phos-tag Acrylamide [11] is commercially available from Wako Pure Chemical Industries (Osaka, Japan). ATP and lithium potassium acetyl phosphate were purchased from Sigma-Aldrich (St. Louis, MO).

### Preparation of recombinant proteins derived from *E*. *coli*

To construct plasmids for overexpression of the cytoplasmic region of hybrid sensor kinases (EvgS, BarA, ArcB, and their Ala-substituted mutants for each phosphorylation site) and for full-length EvgA, the corresponding DNA fragments were prepared by PCR using genome DNA of *E*. *coli* W3110 as a template in conjunction with a set of primer pairs, as described previously [15]. Sequences of primers used in this study were listed in Table 1. After digestion of the PCR-amplified fragments with two types of restriction enzyme, each introducing a single cleavage within one of the primer pairs, the fragments were inserted into a pET21a(+)vector (Merck; Darmstadt, Germany) between the same restriction sites as those used for the preparation of the insert DNAs. Mutations were introduced into the phosphorylation sites of the hybrid sensor kinases by using a Quick-Change Site-Directed Mutagenesis Kit (Stratagene; La Jolla, CA). The sequence was confirmed by using an ABI PRISM 310 Genetic Analyzer (Applied Biosystems; Foster City, CA). Each constructed plasmid was transformed into *E*. *coli* BL21(DE3) or BL21(DE3)pLysS. The host cells were grown in Luria-Bertani (LB) broth at 37°C, and the targeted proteins were overexpressed by induction with isopropyl β-d-1-thiogalactopyranoside. N-Terminal proteins tagged on histidine residues were purified by using nickel—NTA agarose (Qiagen; Hilden, Germany). The purified proteins were stored in 10 mM Tris—HCl (pH 8.0) containing 50% (v/v) glycerol at –20°C.

**Table 1:** PCR primers used in this study.

Primer	Sequence (5'–3')	Function
EvgS (truncated)_F	TGGGGATTCTACGGATCCCGCTCAGTTCGT	Amplification of truncated EvgS
EvgS (truncated)_R	ATTGTGGGAGCCGCGGCCGCGTCATTTTTC	Amplification of truncated EvgS
EvgS (H721A)_ F	TCTGGCAACGATGAGTGCCGAAAATAAGAACACCA	Mutagenesis
EvgS (H721A)_R	TGGTGTTCTTATTTTCGGCACTCATCGTTGCC	Mutagenesis
EvgS (D1009A)_F	GATCTGCTTATTACTGCCGTTAATATGCCGAA	Mutagenesis
EvgS (D1009A)_R	TTCGGCATATTAACGGCAGTAATAAGCAGATC	Mutagenesis
EvgS (H1137A)_F	TTCCATCAGTGTATTGCCCGCATCCACGGTGC	Mutagenesis
EvgS (H1137A)_R	GCACCGTGGATGCGGGCAATACACTGATGGAA	Mutagenesis
EvgS (F577S)_ F	GAAAACCAAATATCATCCCGAAAAGCACTCTC	Mutagenesis
EvgS (F577S)_R	GAGAGTGCTTTTCGGGATGATATTTGGTTTTC	Mutagenesis
EvgA_F	CAAAGGGAAGGATCCATGAACGCAATAATT	Amplification of EvgA
EvgA_R	AAAAACTTCAGCGGCCGCGCCGATTTTGTT	Amplification of EvgA
BarA (truncated)_F	TTCTATCGGTGGTCGGATCCCAACTGGAGG	Amplification of truncated BarA
BarA (truncated)_R	GGTCTAGCGCGGCCGCTTTTTTAGTGGCTT	Amplification of truncated BarA
BarA (H302A)_F	CTGGCAAATATGTCAGCCGAGCTGCGTACACCA	Mutagenesis
BarA (H302A)_R	TGGTGTACGCAGCTCGGCTGACATATTTGCCAG	Mutagenesis
BarA (D718A)_F	GATTTGATCTTAATGGCTATTCAAATGCCTGAC	Mutagenesis
BarA (D718A)_R	GTCAGGCATTTGAATAGCCATTAAGATCAAATC	Mutagenesis
BarA (H861A)_F	CTGGTTGATTTGATTGCTAAACTGCATGGCAGT	Mutagenesis
BarA (H861A)_R	ACTGCCATGCAGTTTAGCAATCAAATCAACCAG	Mutagenesis
ArcB (truncated)_F	TTCTATCGGTGGTCGGATCCCAACTGGAGG	Amplification of truncated ArcB
ArcB (truncated)_R	GGTCTAGCGCGGCCGCTTTTTTAGTGGCTT	Amplification of truncated ArcB
ArcB (H292A)_F	ATCTCCACCATCAGTGCCGAATTGCGTACACCG	Mutagenesis
ArcB (H292A)_R	CGGTGTACGCAATTCGGCACTGATGGTGGAGAT	Mutagenesis
ArcB (D576A)_F	GACCTGGTGTTGCTGGCTATTCAGTTGCCAGAT	Mutagenesis
ArcB (D576A)_R	ATCTGGCAACTGAATAGCCAGCAACACCAGGTC	Mutagenesis
ArcB (H717A)_F	ATTGTTGAGGAAGGAGCTAAAATTAAAGGTGCG	Mutagenesis
ArcB (H717A)_R	CGCACCTTTAATTTTAGCTCCTTCCTCAACAAT	Mutagenesis

### *In vitro* phosphorylation assay

Autophosphorylation of EvgS and the phosphorelay reaction of EvgS/EvgA (each at 0.4 mg/mL) were performed in 0.30 M Tris—HCl (pH 8.0) containing 50 mM KCl, 10 mM MgCl_2_, 1–40 mM ATP or 370 kBq of [γ-^32^P]-ATP) at 25°C. Autophosphorylations of BarA and ArcB (each at 0.4 mg/mL) were performed in 50 mM Tris—HCl (pH 8.0) containing 25 mM KCl, 5.0 mM MgCl_2_, 10 mM dithiothreitol, and 10 mM ATP at 25°C. Acetyl phosphate-dependent autophosphorylations of EvgS and EvgA (each at 0.4 mg/mL) were performed in 0.30 M Tris—HCl (pH 8.0) containing 50 mM KCl, 10 mM MgCl_2_, and 40 mM acetyl phosphate at 25°C. The reactions were carried out for the appropriate times, then terminated by adding a half volume of 3× sample-loading buffer for SDS-PAGE, consisting of 195 mM Tris—HCl (pH 6.8), 3.0% (w/v) SDS, 30% (v/v) glycerol, 15% (v/v) 2-sulfanylethanol, and 0.10% (w/v) bromophenol blue (BPB). Sample solutions were not boiled before electrophoresis.

### Phos-tag SDS-PAGE

Electrophoresis was usually performed at 30 mA/gel and room temperature by using a 1-mm-thick, 9-cm-wide, and 9-cm-long gel on an AE-6500 PAGE apparatus (Atto; Tokyo, Japan). The separating gel (6.3 mL) consisted of 6–7% (w/v) polyacrylamide and 375 mM Tris—HCl buffer (pH 8.8), and the stacking gel (1.8 mL) consisted of 4% (w/v) polyacrylamide and 125 mM Tris—HCl buffer (pH 6.8). Phos-tag Acrylamide (25 μM) and two equivalents of MnCl_2_ (50 μM) were added to the separating gel before polymerization. An acrylamide stock solution was prepared containing a 29:1 mixture of acrylamide and *N*,*N*'-methylenebisacrylamide. The running buffer consisted of 192 mM glycine and 25 mM Tris containing 0.10% (w/v) SDS. The electrophoresis was continued until the BPB dye reached the bottom of the separating gel. After electrophoresis, the gels were stained with a solution of colloidal Coomassie Brilliant Blue G-250 (cCBB). The cCBB solution was prepared as follows. (i) Al_2_(SO_4_)_3_ 14–18H_2_O (50 g) was dissolved in distilled water (800 mL). (ii) EtOH (100 mL) was added with stirring. (iii) CBB G-250 (0.20 g) was dissolved with stirring. (iv) 85% phosphoric acid (24 mL) was added with stirring. (v) Finally, the solution was diluted to 1 L with distilled water. Densitometric analyses of the stained gels were performed by using Atto Densitograph software. For autoradiography, the gel was dried at 80°C under vacuum, and then exposed on an imaging plate for 1–2 h. The imaging plate was scanned by using an FLA 5000 image analyzer (Fujifilm; Tokyo, Japan).

### *In vivo* EvgS phosphorylation

Full-length EvgS was expressed using the pBADevgS plasmid having an *evgS* gene inserted downstream of an arabinose promoter [16]. By using the pBADevgS plasmid as a template, an F577S mutation in the *evgS* gene was introduced to construct the pBADevgS1 plasmid [17]. These plasmids were transformed into *E*. *coli* MG1655 *evgS ydeP-lacZ* [16]. The strains were grown at 37°C in LB broth containing 0.1 mg/mL ampicillin until the OD_600_ reached 0.3, and then l-arabinose was added to a concentration of 0.02% (w/v) to induce expression of EvgS or EvgS1. The cells were further incubated for 2 h then collected and suspended in 10 mL of warmed M9 medium (6 g Na_2_HPO_4_, 3 g KH_2_PO_4_, 0.5 g NaCl, and 1 g NH_4_Cl per liter, with the addition of 0.1 mM CaCl_2_, 1 mM MgSO_4_, 0.2% (w/v) glucose, 0.0001% (w/v) vitamin B_1_, and 0.004% (w/v) casamino acids, pH 7.0) containing 0.1 M KCl, 0.02% (w/v) l-arabinose, and 0.1 mg/mL ampicillin. The cells were then incubated for a further 1 h. For acid stimulation, 60 μL of 6 M aqueous HCl was added beforehand to 10 mL of the above M9 medium to adjust the pH to 5.5. For the β-galactosidase assay, 20 μL of culture was mixed with 180 μL of an assay buffer consisting of 60 mM Na_2_HPO_4_, 40 mM NaH_2_PO_4_, 10 mM KCl, 1 mM MgSO_4_, 50 mM 2-sulfanylethanol, 0.001% (w/v) SDS, and 0.005% (v/v) trichloromethane. The β-galactosidase reactions were initiated by adding 40 μL of 4 mg/mL 2-nitrophenyl d-galactopyranoside and, after 5 min, all reactions were quenched by adding 0.1 mL of 1 M Na_2_CO_3_. Absorbances at 420 and 600 nm (OD_420_ and OD_600_, respectively) were measured, and the activity was calculated as follows: 1 unit = OD_420_/OD_600_ × 1000. For analysis using Phos-tag SDS-PAGE, a 2 mL aliquot of culture was centrifuged and the pelleted cells were washed with 1 mL Tris—HCl (pH 6.8) and then lysed with 0.20 mL of 1× sample-loading buffer for SDS-PAGE containing 5 units of benzonase. The lysates were immediately analyzed by Phos-tag SDS-PAGE or stored at –20°C; stored lysates were used within two days. After Phos-tag SDS-PAGE, EvgS was detected by Western blotting using an anti-EvgS antiserum, which was prepared in our previous study [16].

## Results

### Separation analysis of the phosphorylated protein species produced in the EvgAS system by using Phos-tag SDS-PAGE

**Fig 1:**
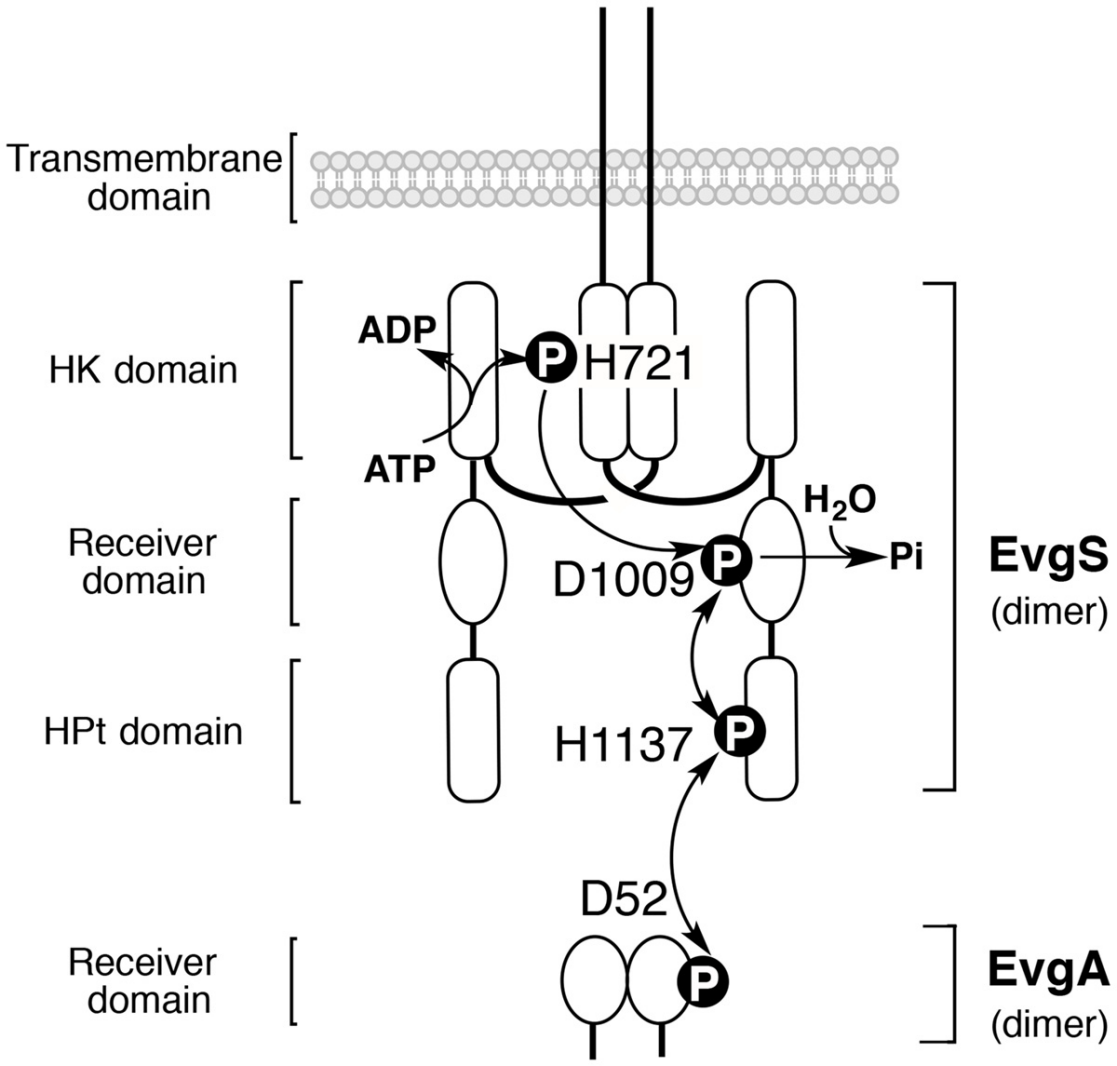
Schematic representation of the EvgAS system. The system is composed of a hybrid sensor kinase (EvgS) and a cytoplasmic response regulator (EvgA). Both EvgS and EvgA function as dimers. Each subunit of EvgS contains an HK domain, a receiver domain, and an HPt domain. The HK domain consists of two subdomains: an ATP-binding subdomain and a dimerization-inducing His-containing subdomain. The ATP-binding subdomain can catalyze the transfer of the γ-phosphate of ATP to the H721 residue in the dimerization-inducing His-containing subdomain of the opposing subunit. The phosphoryl group would be then transferred in a *cis* manner to the D1009 residue of the receiver domain and then onward to the H1137 residue in the HPt domain. Phosphorylated H1137 would serve as a substrate for reversible phosphorylation of D52 in the receiver domain of EvgA.

An EvgAS system is a two-component regulatory system derived from *E*. *coli*., which consists of a hybrid sensor kinase, EvgS and its cognate response regulator, EvgA. The EvgAS system confers acid resistance to the bacterial cell [16, 18], and EvgS and EvgA function as dimers. A schematic diagram of the EvgAS system could be illustrated as shown in Fig 1, through reference to a homologous BvgAS system in *Bordetella pertussis* [19–21]. At its N-terminus, each EvgS subunit is predicted to have a single transmembrane domain, each of which is connected to a sequence consisting of an HK domain, a receiver domain, and an HPt domain. The HK domain consists of two subdomains: an ATP-binding subdomain and a dimerization-inducing His-containing subdomain. The ATP-binding subdomain, when active, can catalyze the transfer of the γ-phosphate of ATP to His 721 (H721) in the dimerization-inducing His-containing subdomain of the opposing subunit. This phosphoryl group would be then transferred in a *cis* manner to Asp 1009 (D1009) in the receiver domain, and from there to His 1137 (H1137) in the HPt domain. The transferred phosphoryl group to the HPt domain could be often returned to the receiver domain during the autophosphorylation reaction. Phosphorylated H1137 would serve as a substrate for reversible phosphorylation of Asp 52 (D52) in the receiver domain of EvgA.

**Fig 2:**
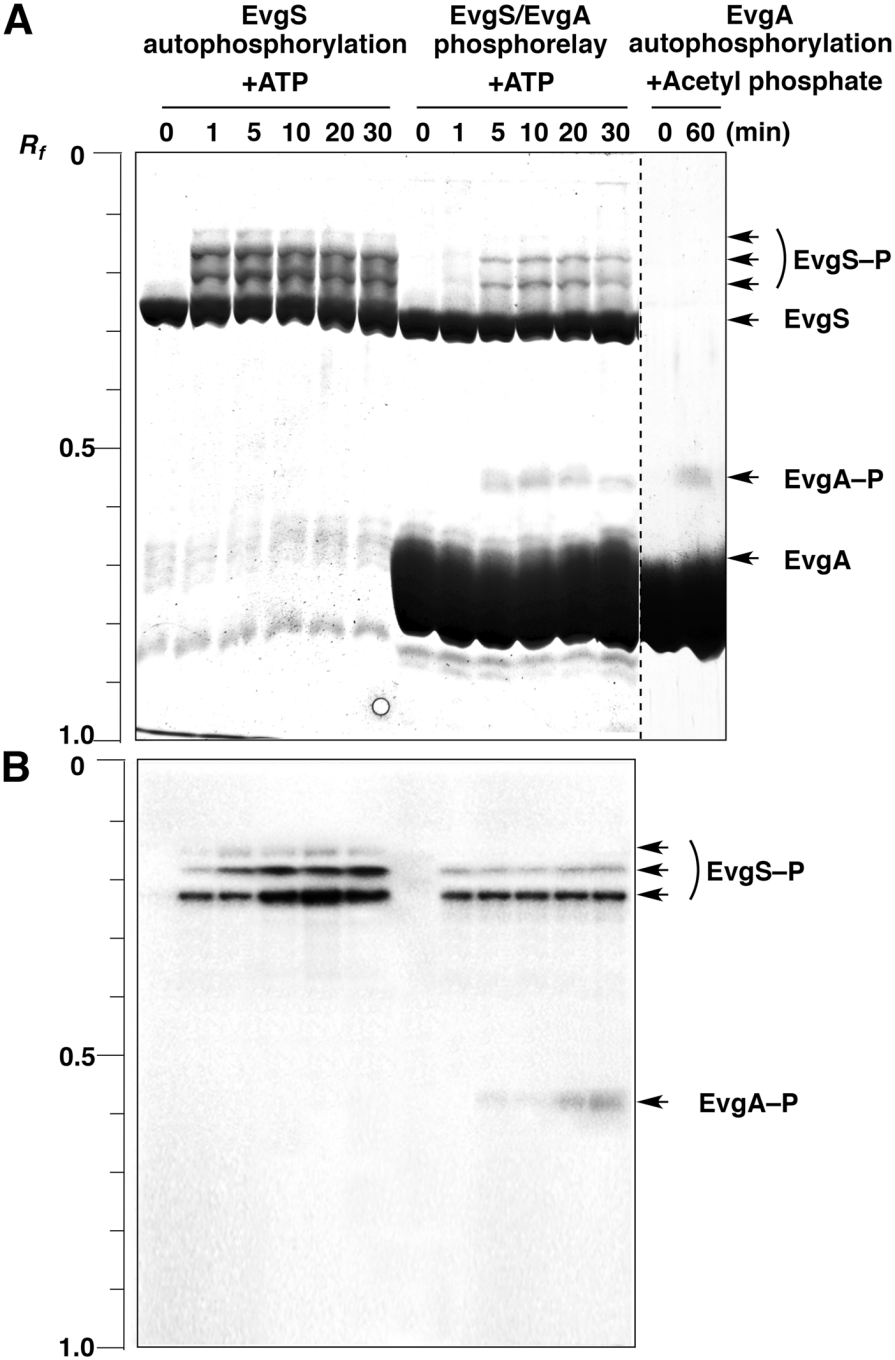
Profiling of EvgS autophosphorylation, the EvgS/EvgA phosphorelay, and EvgA autophosphorylation by using Phos-tag SDS-PAGE. (A) The EvgS autophosphorylation and EvgS/EvgA phosphotransfer reactions were performed in the presence of 30 mM ATP. The EvgA autophosphorylation reaction was performed in the presence of 40 mM acetyl phosphate. Gels were stained with cCBB. The incubation times for the reactions are shown above each lane. Each lane contained 2 μg of EvgS or EvgA. (B) The EvgS autophosphorylation and EvgS/EvgA phosphotransfer reactions were performed in the presence of 370 kBq of [γ-^32^P]-ATP. The gel was subjected to autoradiography.

On the basis of the scheme of the multistep phosphorelay on the EvgAS system shown in Fig 1, Phos-tag SDS-PAGE was used to determine the changes over time from 0 to 30 min in the composition of phosphorylated protein species during the course of the autophosphorylation reaction of EvgS *in vitro* and of the phosphoryl-transfer reaction between EvgS and EvgA *in vitro* (Fig 2A). The autophosphorylation reaction of EvgS proceeded rapidly in the presence of 30 mM ATP, and bands of three phosphorylated forms (EvgS—P) were observed, even at 1 min. The three upshifted bands were presumed to correspond to three forms of EvgS phosphorylated at the His 721 residue (H721) on the HK domain, at the Asp 1009 residue (D1009) on the receiver domain, or at the His 1137 residue (H1137) on the HPt domain, respectively (see Fig 1). On the other hand, when we added EvgA in the expectation that an intermolecular phosphoryl-transfer reaction from EvgS to EvgA might occur, the three phosphorylated forms of EvgS were detected at lower densities than those observed in the absence of EvgA (that is, in the autophosphorylation reaction of EvgS). In this case, a phosphorylated form of EvgA (EvgA—P) was also observed as a single upshifted band at relatively low density.

Because the Asp residue on the receiver domain of various bacterial response regulators, including BvgA of *B*. *pertussis* that is homologous to EvgA, can be directly autophosphorylated by treatment with acetyl phosphate as a phosphoryl donor [22–24], we performed the autophosphorylation reaction of EvgA in the presence of 40 mM acetyl phosphate and compared the mobility of EvgA—P obtained as described above with that of EvgA—P separately prepared with acetyl phosphate. Bands for both samples of EvgA—P in the Phos-tag gels showed the same mobility. This confirmed that the form of EvgA phosphorylated at the Asp residue (D52) on the receiver domain of EvgA was actually produced by acceptance of a phosphoryl group from the HPt domain of EvgS in the reaction where both EvgS and EvgA were present. Note that because the contrast of the whole image was adjusted to permit visualization of the weak EvgA—P bands, the exposure intensity of the remaining bands exceeded the range in which quantitative accuracy is maintained.

The results of autoradiography with [γ-^32^P]-ATP (Fig 2B) confirmed that the upshifted bands of EvgS and EvgA observed in Fig 2A are the corresponding phosphorylated forms. To determine the stoichiometry of phosphate incorporation into EvgS, the ratios of the ^32^P signal intensity to the density of gel staining for the three upshifted bands of EvgS in the autophosphorylation reaction were evaluated by densitometric analysis, as described previously [13]. The obtained ratios were almost equal, indicating that the three upshifted bands have the same number of phosphoryl groups per molecule of protein (S1 Fig). The densitometric result is consistent with our estimation that the three bands correspond to the EvgS forms monophosphorylated at each phosphorylation site (H721, D1009, or H1137). During the autophosphorylation reaction or the intramolecular phosphoryl-transfer reaction of EvgS, therefore, it should be unlikely that a phosphorylated form having two or more phosphoryl groups is produced in each EvgS subunit.

### Identification of the three phosphorylated forms of EvgS

**Fig 3:**
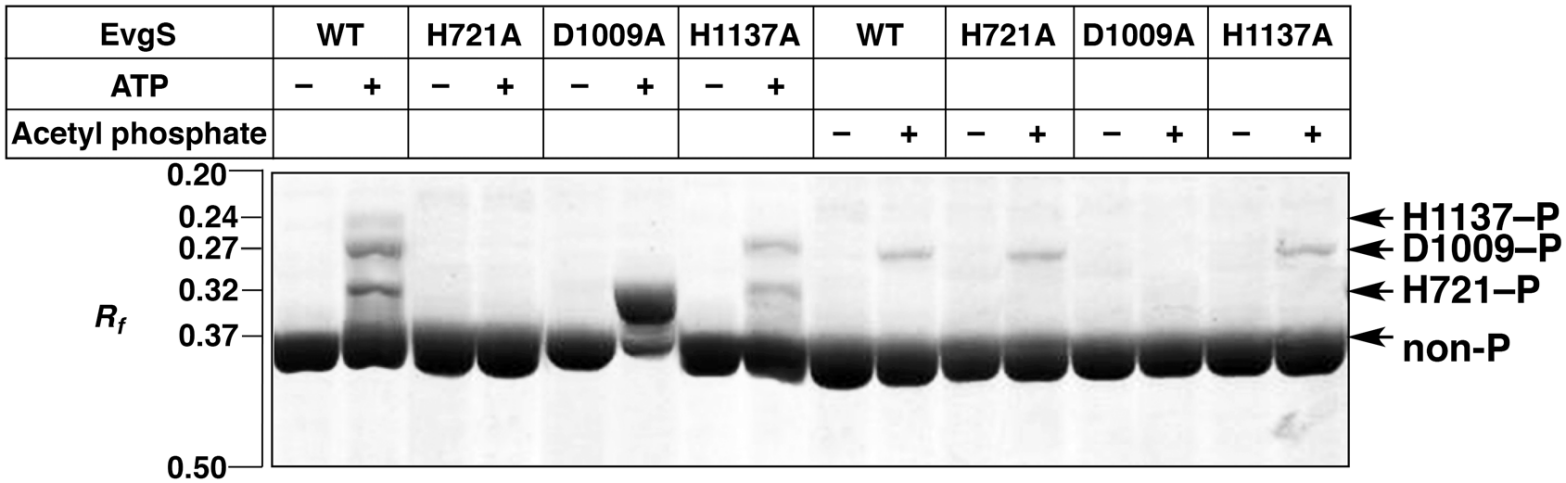
Identifications of the phosphorylation sites of the three phosphorylated forms of EvgS by means of Ala-substituted site-specific mutagenesis. Autophosphorylation reactions of wild-type EvgS (WT) and its three Ala-substituted mutants (H721A, D1009A, and H1137A) were performed in the presence of 30 mM ATP or 40 mM acetyl phosphate for 30 min. Each lane contained 2 μg of the appropriate EvgS protein. Bands for the three site-specific phosphorylated forms were assigned and are shown by arrows on the right-hand side of the panel.

To identify the sites of phosphorylation of the three phosphorylated forms of EvgS observed in Fig 2, the band mobilities of the corresponding phosphorylated forms in Phos-tag SDS-PAGE were compared in autophosphorylation reactions using wild-type EvgS and the three Ala-substituted mutants: H721A, D1009A, and H1137A (Fig 3). When ATP was used as a phosphoryl donor, three types of upshifted bands at *R*_*f*_ = 0.24 (low-mobility form), 0.27 (medium-mobility form), and 0.32 (high-mobility form), respectively, similar to those shown in Fig 2, were detected for wild-type EvgS, whereas no upshifted band was detected for the H721A mutant (see left-hand side of Fig 3). The D1009A mutant showed a single strong band at the position of the high-mobility form and a much weaker band of nonphosphorylated EvgS (*R*_*f*_ = 0.37), whereas the medium-mobility and low-mobility forms were not detected. The H1137A mutant provided two upshifted bands at the positions of the high-mobility and medium-mobility forms, whereas the low-mobility form was not detected. For these results and the phosphorylation scheme proposed (see Fig 1), we assigned the low-mobility, medium-mobility, and high-mobility bands to the phosphorylated forms H1137–P (with a phosphorylated H1137 residue), D1009–P (with a phosphorylated D1009 residue), and H721–P (with a phosphorylated H721 residue), respectively (indicated by the arrows on the right-hand side of Fig 3). Furthermore, we confirmed these assignments by an analysis of the autophosphorylation reaction using acetyl phosphate as a phosphoryl donor: the wild type, the H721A mutant, and the H1137A mutant shows a single upshifted band at the position of the medium-mobility form, whereas no upshifted band was detected for the D1009A mutant. These results are consistent with selective autophosphorylation of the Asp residue on the receiver domain of hybrid sensor kinases by acetyl phosphate, as described previously [2].

It is interesting to note that in the ATP-dependent autophosphorylation reaction, the D1009A—P phosphorylated form (that is, the high-mobility form of the D1009A mutant with a phosphorylated H721 residue) was produced in excess in comparison with the wild type. This phenomenon was confirmed as strong radioactivity by autoradiography using [γ-^32^P]-ATP (S2 Fig).

### Kinetics of EvgS phosphorylation

**Fig 4:**
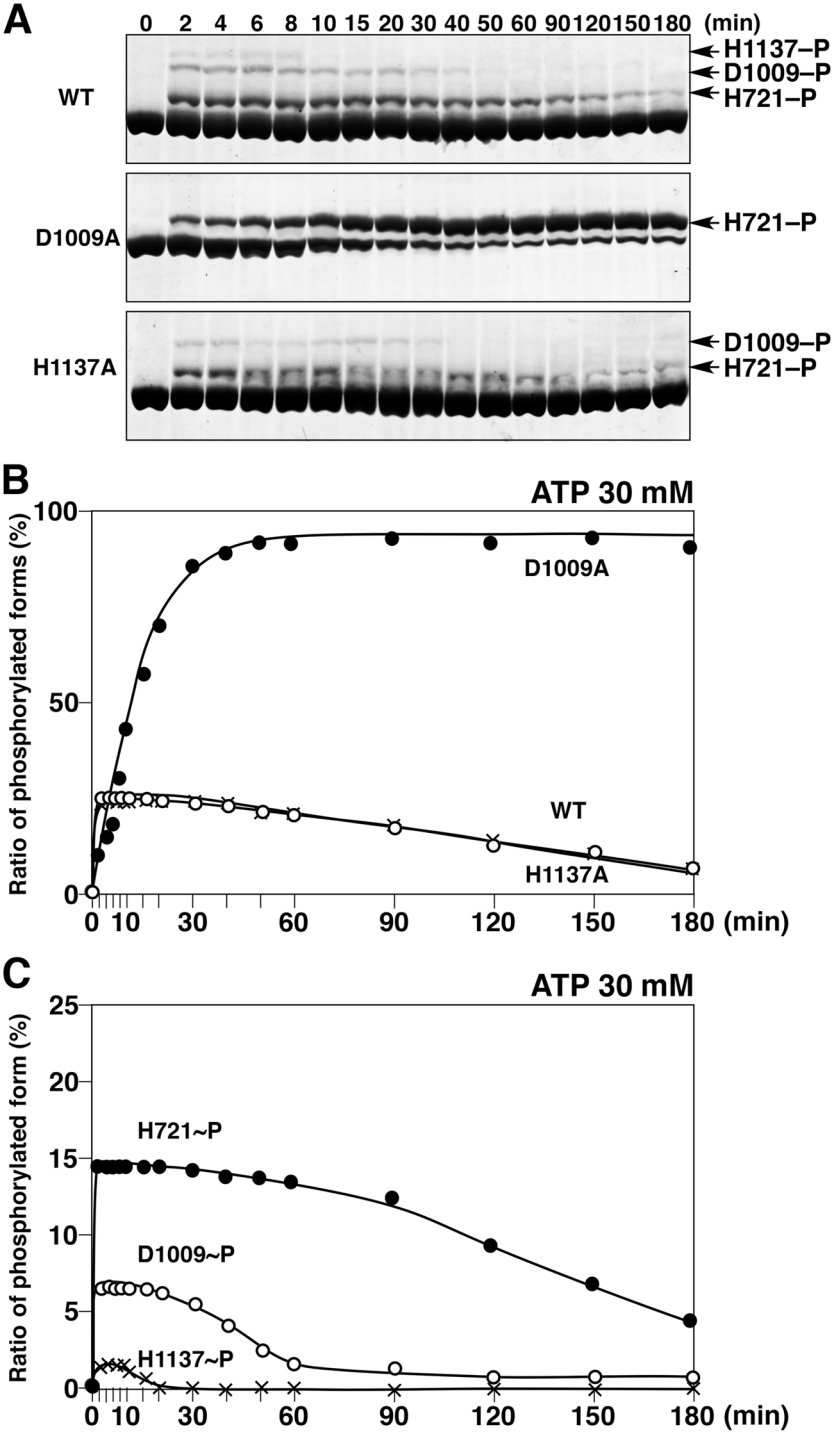
Time-dependent changes in levels of phosphorylation of wild-type EvgS (WT) and the two mutants D1009A and H1137A. (A) Autophosphorylation reactions of WT, D1009A, and H1137A were performed in the presence of 30 mM ATP and analyzed by Phos-tag SDS-PAGE. The incubation times are shown above each lane. Each lane contained 2 μg of protein. (B) The values of the ratios of the phosphorylated forms to the total proteins were calculated by densitometry and are plotted versus the reaction times. (C) The values of the ratios for each phosphorylated form derived from the WT to the total proteins were calculated and are plotted versus the reaction times. Each plot was gained as average of three independent experiments using the same sample. Standard deviations were within almost 20%.

To elucidate the kinetics of phosphorylation of EvgS and its mutants in more detail, we tracked changes in the compositions of phosphorylated protein species during the course of their autophosphorylation reactions over time from 0 to 180 min. The reaction products were analyzed by Phos-tag SDS-PAGE (Fig 4A), and the ratio of the total phosphorylated forms to the total EvgS proteins was calculated by densitometric analysis and plotted against the reaction time (Fig 4B). The phosphorylation reactions were performed in the presence of 30 mM ATP. The autophosphorylation reaction of wild-type EvgS quickly reached a steady state with a maximum ratio of all the phosphorylated forms calculated to be approximately 25%; this ratio then gradually decreased after 10 min. As shown in Fig 1, it is widely accepted that the receiver domain of EvgS has activities of transferring and hydrolyzing a phosphoryl group on the D1009 residue. Hence, the steady state of wild-type EvgS observed at around 10 min is the result of competition between phosphorylation and dephosphorylation of EvgS. The gradual decrease in the phosphorylation ratio after 10 min is probably caused by ATP depletion through phosphate hydrolysis. As for the D1009A mutant, the autophosphorylation ratio reached a plateau value of more than 95% within 60 min, and the value was maintained until 180 min. On the other hand, the autophosphorylation reaction of the H1137A mutant proceeded in almost the same manner as the wild-type protein. These results suggest that the phosphoryl group on the Asp residue (D1009) in the receiver domain is hydrolyzed in preference to the His residue (H721) in the HK domain or the His residue (H1137) in the HPt domain. The ratios of the respective phosphorylated forms (H721–P, D1009–P, and H1137–P) produced in the autophosphorylation reaction of wild-type EvgS to the total EvgS proteins were calculated and plotted against the reaction time (Fig 4C). At 1–10 min of the reaction, the maximum ratios of the H721–P and D1009–P phosphorylated forms were calculated to be approximately 15% and 7%, respectively, whereas that of the H1137–P phosphorylated form was as little as 2% or less. This ratio value for the H1137–P phosphorylated form in the wild type explains the results shown in Fig 2, which demonstrated that the number of phosphoryl groups transferred from the HPt domain of EvgS to the receiver domain of EvgA is very low. Collectively, these findings show that most of the phosphoryl groups that are accepted from ATP in the autophosphorylation reaction are released from the EvgS molecule at the Asp residue in the receiver domain before being transferred to the His residue in the HPt domain.

**Fig 5:**
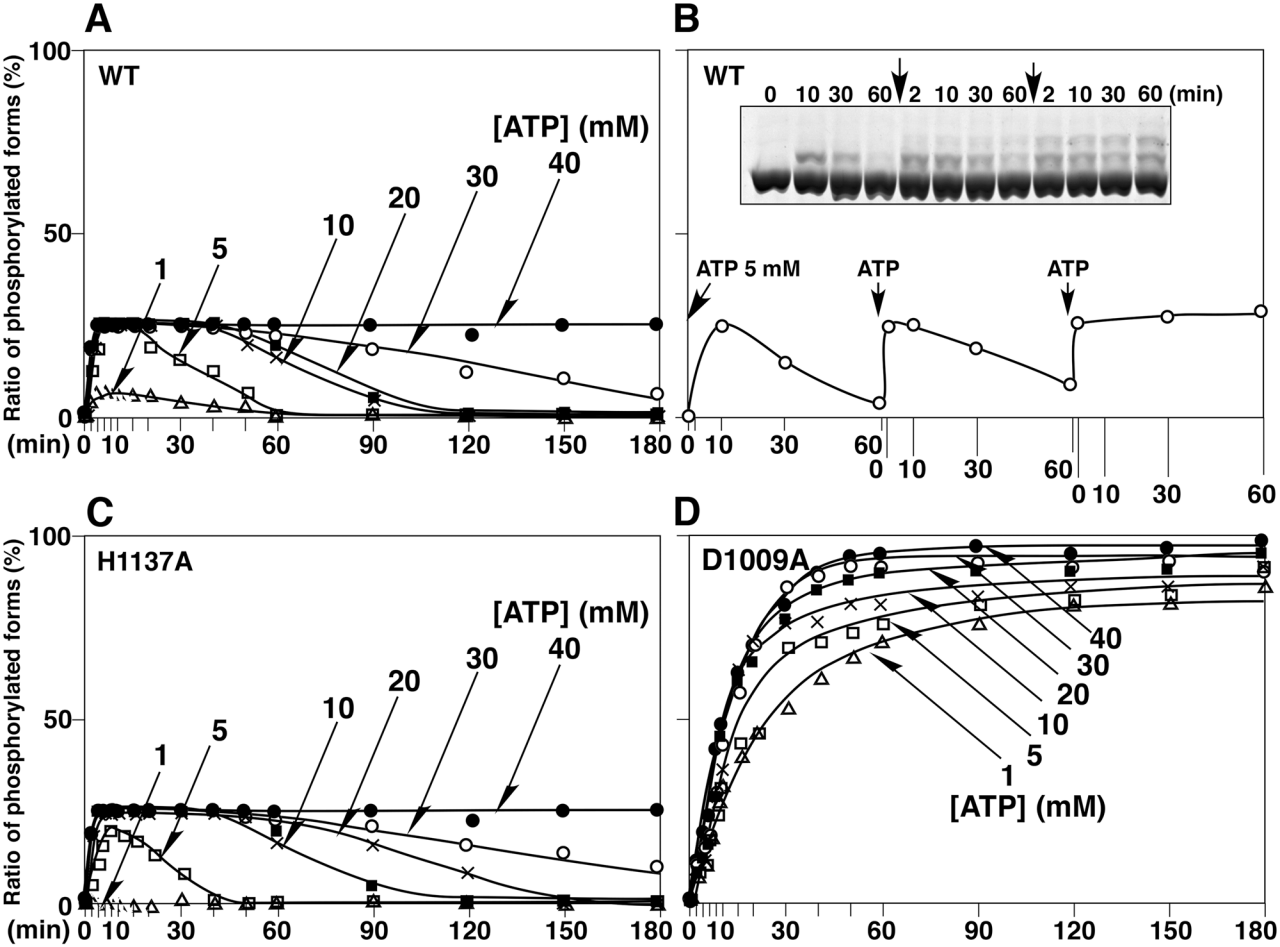
Autophosphorylation reactions of wild-type EvgS (WT) and the two mutants D1009A and H1137A in ATP- and dose-dependent manners. (A) Autophosphorylation reactions of WT were performed in the presence of 0, 1, 5, 10, 20, 30, or 40 mM ATP for 0–180 min. Each reaction was analyzed by Phos-tag SDS-PAGE. The values of the ratios of the phosphorylated forms to the total proteins were determined by densitometry and are plotted versus the reaction times. (B) Autophosphorylation of WT was performed in the presence of 5 mM ATP for 0–60 min. A solution of ATP was then added and the reaction was continued for a further 60 min; this process was repeated once. A 1/20th volume of 100 mM ATP solution was added to the reaction solution (final concentration = 5 mM ATP). Each lane in Phos-tag SDS-PAGE contains 2 μg of protein. (C) Autophosphorylation of the mutant of H1137A was analyzed in the same manner as that of the WT. (D) Autophosphorylation of the mutant of D1009A was analyzed in the same manner as that of the WT. Each plot was gained as average of three independent experiments using the same sample. Standard deviations were within almost 20%.

Next, we compared the time-courses of the autophosphorylation reactions of EvgS and its mutants D1009A and H1137A in the presence of various concentrations of ATP (Fig 5). As in the case of wild-type EvgS at ATP concentrations in excess of 5 mM, the ratio of the total phosphorylated forms quickly reached a maximum level of 25%, and this maximum level was maintained for a certain time in an ATP dose-dependent manner (Fig 5A; the results of Phos-tag SDS-PAGE are shown in S3 Fig). This dependence probably arises as a consequence of ATP depletion during the autophosphorylation reaction. In fact, when the phosphorylation ratio reached a value close to 0% after incubation for 60 min in the presence of 5 mM ATP, addition of a second portion of 5 mM ATP regenerated the phosphorylated species at a similar rate to that at which the phosphorylated species were generated by the first ATP addition (Fig 5B). The time-course patterns of the autophosphorylation reaction of the H1137A mutant were almost the same as those of wild-type EvgS (Fig 5C). As for the D1009A mutant, the rate of autophosphorylation reached a maximum that was depended on the initial ATP concentration of 1–40 mM, and this level of more than 80% was maintained until 180 min, even without any further addition of ATP (Fig 5D).

**Fig 6:**
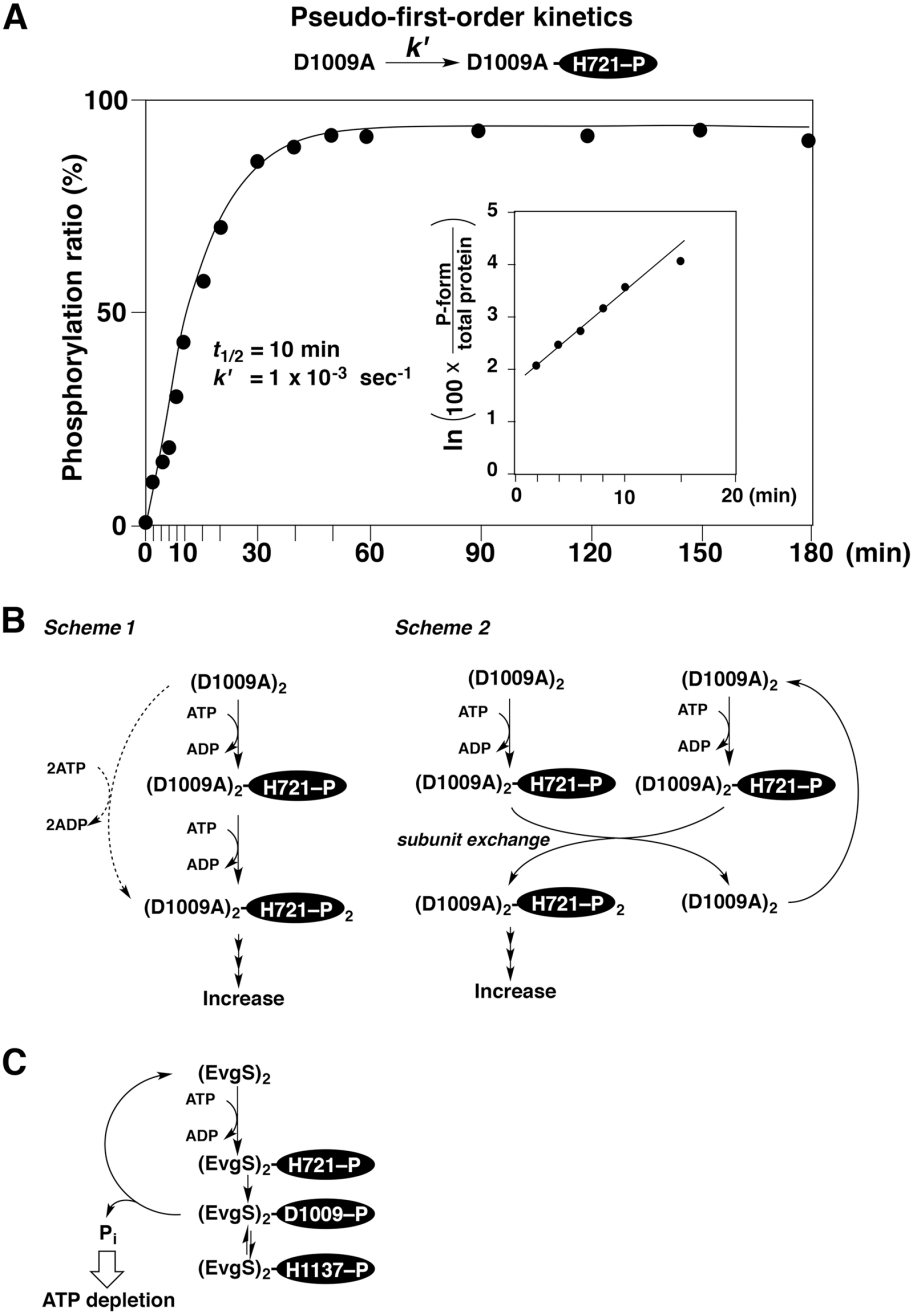
Comparisons of the reaction modes for autophosphorylation of wild-type EvgS and of the mutant of D1009A. (A) Analysis of the kinetics of autophosphorylation of the mutant D1009A. The profile of the phosphorylation ratio (%) versus the reaction times (the same profile as shown in Fig 4B) was fitted to a first-order kinetic model; the half-life time (*t*_1/2_) was 10 min (*k*' = 1 × 10^−3^ s^–1^). In the early stages of the reaction (≤15 min), a plot of the natural logarithm of the phosphorylation ratio (%) against the reaction time formed a straight line. (B) Two schemes (scheme 1 and 2) for the autophosphorylation of the mutant D1009A dimer, (D1009A)_2_, suggested by the kinetic analysis. Scheme 1 is a model of sequential autophosphorylation reactions. Scheme 2 is a model of a flip-flop autophosphorylation reaction followed by an exchange of subunits of the (D1009A)_2_ dimer. (C) Scheme for the autophosphorylation reaction of wild-type EvgS. In the presence of sufficient ATP, a cycle of phosphorylation, intramolecular phosphate transfer, dephosphorylation is repeated.

In the autophosphorylation reaction of the D1009A mutant in the presence of sufficient ATP (30 mM), the time-course of the phosphorylation ratio can be fitted to a pseudo-first-order kinetic model if the results for the first 15 min are used (Fig 6A). The insert in Fig 6A shows a logarithmic plot of the kinetic data, in which the straight line indicates a good fit to the model. The obtained half-life time (*t*_1/2_) of nonphosphorylated D1009A substrate was 10 min (*k*' = 1 × 10^−3^ s^–1^).

On the basis of the results from the kinetic studies, we propose two reaction schemes (scheme 1 and 2) shown in Fig 6B for the hyperphosphorylation of the D1009A mutant. The scheme 1 is a model of sequential autophosphorylation reactions, in which each subunit of the (D1009A)_2_ dimer is sequentially autophosphorylated. As the first step, the H721 residue on one subunit undergoes phosphorylation to form the monophosphorylated species (D1009A)_2_-H721–P. In the next step, the H721 residue on the other subunit is phosphorylated to produce (D1009A)_2_-H721–P_2_, of which final quantity was determined to be more than 95% by Phos-tag SDS-PAGE. To fit the pseudo-first-order kinetic model shown in Fig 6A, the rate for the second phosphorylation of (D1009A)_2_-H721–P must be more than that for the first phosphorylation of (D1009A)_2_. If the second rate is extremely larger than that for the first phosphorylation, the second phosphorylation undergoes simultaneously (see dashed allows in the scheme 1). Previously, a similar model of sequential autophosphorylation reactions has been described for a VicK sensor kinase in *Streptococcus mutans* [25]. In this report, however, the authors have not accepted simultaneous autophosphorylation reactions on both His residues in the HK domain of the dimer.

On the other hand, a model known as the flip-flop autokinase mechanism, in which only one subunit can be phosphorylated in an autophosphorylation reaction, has been described for an EnvZ sensor kinase in *E*. *coli* [26]. Taking the flip-flop mechanism into account, we propose an alternative scheme followed by a rapid exchange of the subunits (see scheme 2 in Fig 6B), which fits the pseudo-first-order kinetic model. Many studies have demonstrated that the facile exchange of subunits between two dimers occurs in some histidine sensor kinases [5, 9, 27–30]. In the scheme 2, therefore, it is also likely that the subunit exchange reaction occurs spontaneously between two monophosphorylated forms of (D1009A)_2_-H721–P. Finally, (D1009A)_2_-H721–P_2_ becomes a dominant species through the scheme 2 under the experimental condition. Thus, in the both schemes 1 and 2, the first phosphorylation of (D1009A)_2_ should be the rate-determining step without the inhibition by ADP or the significant hydrolysis of the H721–P moiety in the kinetic time scale.

The reaction mechanism for wild-type EvgS is more complex as contrasted with that for the D1009A mutant (Fig 6C). The concentrations of the phosphorylated forms H721–P, D1009–P, and H1137–P are probably controlled by multiple reactions, such as the phosphorylation reaction (ATP to H721) in the HK domain, intramolecular phosphoryl-transfer reactions (H721–P to D1009, D1009–P to H1137, and H1137–P to D1009), and the dephosphorylation reaction (D1009–P to D1009 and Pi) in the receiver domain (see Figs 1 and 6C). In this reaction mechanism for the wild-type protein, it is essential that the reaction rate for the phosphate hydrolysis of D1009–P in the receiver domain is much greater than those for the intramolecular phosphoryl-transfer reactions between the receiver and HPt domains, and therefore the production of the H1137–P phosphorylated form is adjusted to an extremely low level, as seen in Fig 4C.

### Phosphorylation profiling of the other hybrid sensor kinases, BarA and ArcB

**Fig 7:**
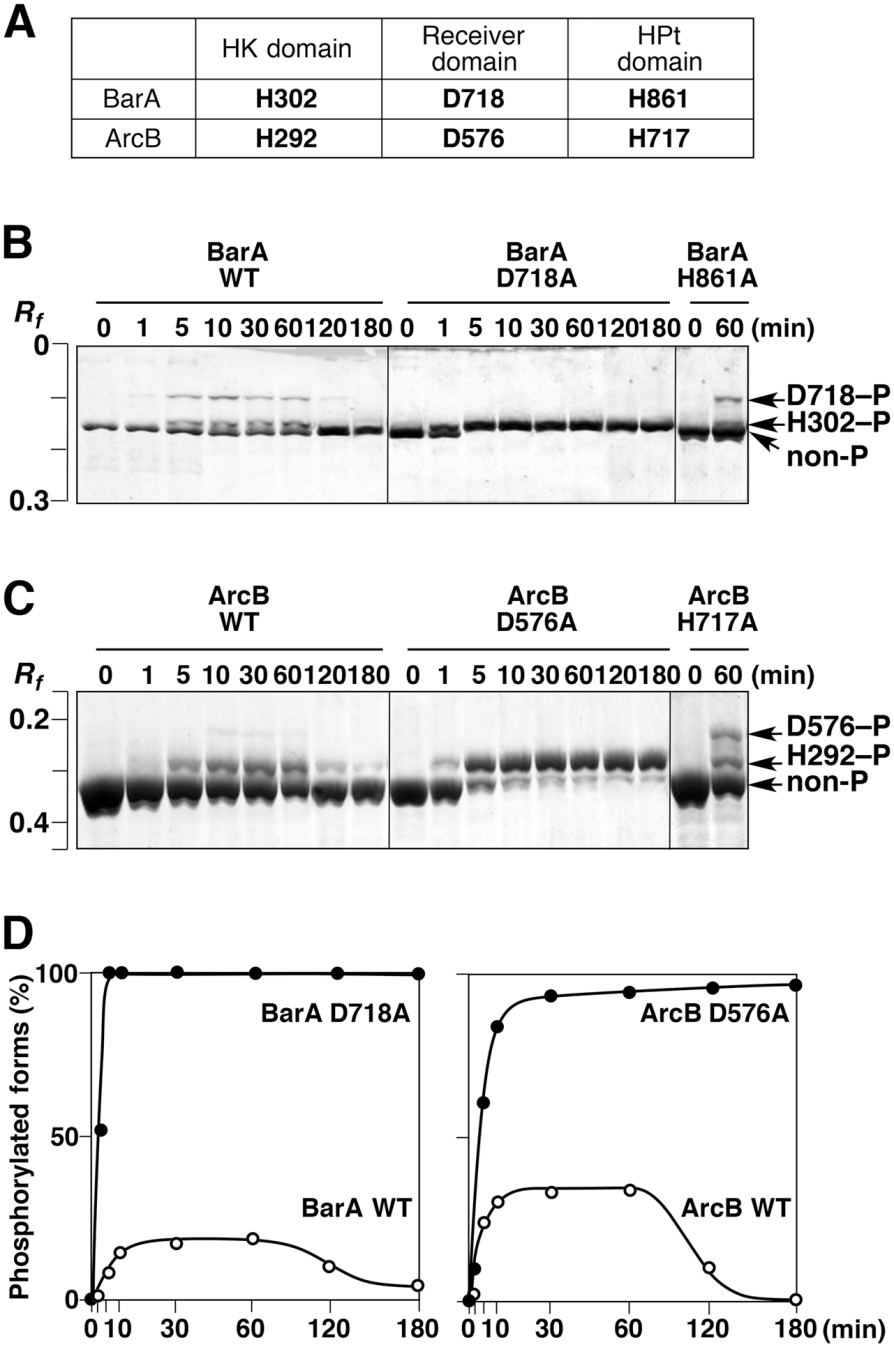
Autophosphorylation reactions of other hybrid sensor kinases, BarA and ArcB. (A) Phosphorylation sites in each domain of BarA and ArcB are shown. (B) Autophosphorylation reactions of wild-type BarA (WT) and the two mutants D718A and H861A were performed in the presence of 10 mM ATP, and the products were then analyzed by Phos-tag SDS-PAGE. The incubation times are shown above each lane. Each lane contained 2 μg of protein. Bands for each of the two site-specific phosphorylated forms were assigned and are shown by arrows on the right-hand side of the panel. (C) Autophosphorylation reactions of wild-type ArcB (WT) and the two mutants D576A and H717A were performed and analyzed in the same manner as BarA in B. (D) Values of the ratio of the phosphorylated forms to the total proteins in B and C were calculated by densitometry and are plotted versus the reaction times. Each plot was gained as average of three independent experiments using the same sample. Standard deviations were within almost 20%.

Mutants of two other hybrid sensor kinases, BarA and ArcB, in which the Asp residue was replaced with Ala, were used to examine whether it would be possible to detect excessive autophosphorylation at the His residue in the HK domain, as observed for the D1009A mutant of EvgS. The phosphorylation sites on each domain of BarA and ArcB are shown in Fig 7A [8, 9]. Two upshifted bands corresponding to phosphorylated forms were observed in a Phos-tag SDS-PAGE analysis of the temporal changes in the composition of phosphorylated protein species during the course of the autophosphorylation reaction of wild-type BarA in the presence of 10 mM ATP (Fig 7B). Only one upshifted strong band was observed in an analysis of the time-course of the autophosphorylation of the D718A mutant, and two upshifted bands with the same mobilities as those for the wild type were observed in an analysis of the autophosphorylation reaction of the H861A mutant. The Ala-substituted-mutagenesis experiments permitted the identification of the phosphorylation sites for each of the bands, as indicated by the arrows on the right-hand side of Fig 7B. Because of its low level of phosphorylation, the H861–P form with a phosphorylated His residue in the HPt domain was not detectable. In the presence of 10 mM ATP, the D718A mutant was stoichiometrically phosphorylated, and the ratio of the H302–P phosphorylated form was close to 100% after 5 min, and this was maintained up to 180 min (Fig 7D). Thus, phosphorylation at the His residue in the HK domain is enhanced by the Ala substitution at the Asp residue in the receiver domain, as seen for the D1009A mutant of EvgS. As for wild-type BarA, the maximum ratio of the total phosphorylated forms was approximately 15%, and this value decreased gradually after 60 min under the same experimental conditions (see Fig 7D).

Next, we conducted a similar mutagenesis experiment for ArcB, in which excessive phosphorylation was once more detected at the H292 residue in the HK domain of the D576A mutant (Fig 7C and 7D). These findings suggest that the mechanism for regulating the quantity of phosphoryl groups transferred to the response regulator, identified by phosphorylation profiling of the EvgAS system using Phos-tag SDS-PAGE, might be a well-conserved mechanism common to other hybrid sensor kinases.

### *In vivo* EvgS phosphorylation assay

On the basis of our *in vitro* results for phosphorylation profiling of the phosphoryl-transfer reaction in the EvgAS system, we attempted to identify the *in vivo* mechanism for the regulation of the quantity of phosphoryl groups to be transferred for EvgS. We expressed the entire length of the EvgS protein in an *evgS*-deleted reporter strain (MG1655 *evgS ydeP-lacZ*), as described previously [16]. In this strain, *evgS* has been deleted and *lacZ* has been inserted immediately downstream of *ydeP*, a regulon of EvgA. In addition, we constructed pBADevgS1, which expressed constantly activated EvgS (EvgS1), by introducing the F577S mutation into pBADevgS [17]. The EvgS1 protein might be able to undergo autophosphorylation at all times, as well as constantly transferring a phosphoryl group to EvgA. Moreover, the mutations of H721A, D1009A, and H1137A were introduced into both pBADevgS and pBADevgS1. The expression of EvgS, EvgS1, and their Ala-substituted mutants was induced by arabinose in an LB medium. Because the EvgAS system can be activated by acidic pH condition in the presence of alkali metals [16], the bacterial cells were transferred to an M9 medium containing 0.1 M KCl at a pH value adjusted to either 7 or 5.5 with hydrochloric acid and then incubated for 1 h.

**Fig 8:**
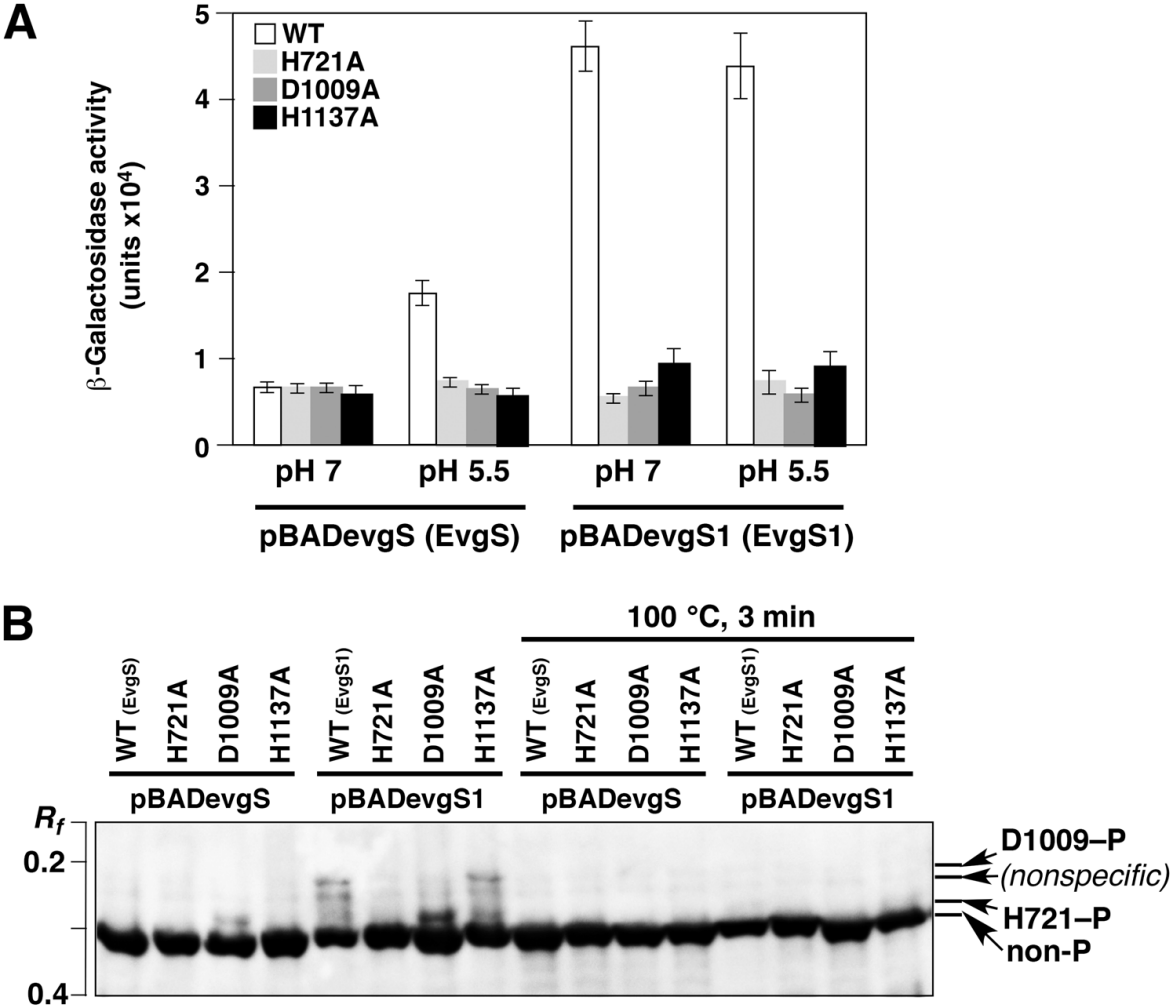
*In vivo* analysis of EvgS phosphorylation. (A) β-galactosidase assays to confirm the arabinose-induced activities of EvgS, EvgS1, and their mutants H721A, D1009A, and H1137A, expressed by a pBAD vector. When using *E*. *coli* MG1655 *evgS ydeP-lacZ* as a host cell, the activation of the EvgAS system induces transcription of *lacZ*, which controls the promoter of *ydeP* (evgA regulon). For both EvgS and EvgS1, the proteins without the Ala substitution at each phosphorylation site are described as WT. Data were represented by mean values and standard deviation bars obtained from three independent experiments. (B) *In vivo* profiling of autophosphorylation reactions of EvgS, EvgS1, and their mutants by using Phos-tag SDS-PAGE followed by Western blotting. Half of each lysate samples was boiled for 3 min to hydrolyze phosphoryl groups on His and Asp residues. Each lane contains 20 μg of the crude cellular protein. Bands for each of the two site-specific phosphorylated forms were assigned and are shown by arrows on the right-hand side of the panel.

The activities of EvgS or EvgS1 inside the cells were examined by using the activity of β-galactosidase as an indicator (Fig 8A). For wild-type EvgS, β-galactosidase activity was significantly observed at pH 5.5. For EvgS1, the activity of β-galactosidase was higher than that of the wild-type EvgS at pH values of both 7 and 5.5. The strains expressing EvgS, EvgS1, and their mutants, incubated at pH 5.5, were separately collected and lysed by using 1× sample-loading buffer for SDS-PAGE, and then subjected to Phos-tag SDS-PAGE (Fig 8B). The bands of these proteins were visualized by Western blotting with the anti-EvgS antiserum. For wild-type EvgS, although significant activity of β-galactosidase was observed (Fig 8A), the density of bands corresponding to the phosphorylated forms of EvgS was not sufficiently high to permit their detection in Phos-tag SDS-PAGE. In contrast, for the EvgS D1009A mutant, a single upshifted band corresponding to the H721–P phosphorylated form was sufficiently strong to permit detection. For EvgS1, two bands corresponding to the H721–P and the D1009–P phosphorylated forms were clearly observed for the wild-type EvgS and the EvgS H1137A mutant. These results imply a constant and enhanced autophosphorylation of EvgS1. The enhancement of the autophosphorylation reaction of EvgS1 is consistent with the results for the β-galactosidase activity in EvgS1 (see Fig 8A). In addition, for the EvgS1 D1009A mutant, a single upshifted band corresponding to the H721–P phosphorylated form was detected, and the density of this band was higher than that detected for the EvgS1 protein or the EvgS1 H1137A mutant.

These findings indicate that phosphorylation of the HK domain is significantly enhanced by deletion of the Asp residue in the receiver domain of a hybrid sensor kinase. Whereas the ratio of phosphorylated forms of the EvgS D1009A mutant reached a value in excess of 95% in the *in vitro* assay, the ratio of the phosphorylated forms remained at a low level in the *in vivo* assay. It has been reported that the concentration of ATP in the single living cell of *E*. *coli* is 1.54 mM on the average [31]. Taking the ATP concentration (more than 1 mM) and our *in vitro* result shown in Fig 5D into consideration, it is probable that the ratio of the phosphorylated forms of the EvgS D1009A mutant would reach a value close to 80% or more in the *in vivo* assay. However, the phosphorylation level of the mutant *in vivo* was actually lower than that *in vitro*, which is possibly due to a slower exchange of the subunits by the restricted movement of EvgS in the cell membrane. Thus, the mechanism of *in vivo* phosphorylation reaction of the D1009A mutant seems to fit the scheme 2 in Fig 6B.

All the upshifted bands disappeared upon heat treatment of the lysates at 100°C for 3 min, showing that the phosphoryl groups on the His and the Asp residues are chemically unstable and confirming that the observed bands corresponded to phosphorylated forms of EvgS and EvgS1.

## Discussion

We separated various site-specifically phosphorylated species of hybrid sensor kinases by using Phos-tag SDS-PAGE and we examined the rates of the phosphoryl-transfer reactions of the enzymes qualitatively and quantitatively. The results elucidated the previously unknown kinetics of the phosphoryl-transfer reaction of the hybrid sensor kinase EvgS. Phosphorylation at the His residue in the HK domain is significantly enhanced in a mutant form of the enzyme in which the Asp residue in the receiver domain is replaced by Ala. The autophosphorylation reaction of the D1009A mutant in the presence of sufficient ATP fitted a pseudo-first-order kinetic model, implying the absence in this mutant of mechanisms for negative regulation of the autophosphorylation level at the His residue in the HK domain. In other words, we showed that the level of autophosphorylation in the HK domain can be negatively regulated by the receiver domain mainly. In addition, the level of phosphorylation at the Asp residue in the receiver domain of the wild-type EvgS was reduced to an extremely low level by the phosphatase activity of the receiver domain. The rate of hydrolysis of the phosphoryl group was greater than the rate of transfer of a phosphoryl group to the HPt domain, and this served to keep the level of phosphorylation in the HPt domain at an even lower level. This negative regulation mechanism by the receiver domain might be common to other hybrid sensor kinases, BarA and ArcB. On the other hand, it has been reported that SixA was identified as a regulator protein of the phosphorylation level of the HPt domain of ArcB during the multistep phosphorelay [32]. The SixA protein exhibits a phosphatase activity toward the phosphorylated His residue in the HPt domain of ArcB. The *trans*-regulation of phosphorylation of ArcB by SixA bears a resemblance to the regulation mechanism by the receiver domain elucidated in this study, from the point of view that the nature of regulation is a phosphatase activity of the regulator.

In the *in vitro* experiments, nearly all the phosphoryl groups were delivered to the aqueous environment, resulting in ATP depletion. It is therefore likely that the ratios of the three phosphorylated forms of the wild-type EvgS are regulated by both the phosphotransferase activity and the phosphatase activity of the receiver domain. In the presence of EvgA, the number of phosphoryl groups relayed to the response regulator was less than 2% of those transferred from ATP to the HK domain under the experimental conditions. Although the waste of ATP by the receiver domain seems to be biologically unfavorable, it must be essential for precise control of the concentration of the activated response regulator at an appropriate level and/or to a signal-off mechanism.

Furthermore, the demonstration *in vivo* of the validity of the mechanism of phosphorelay control proposed as a result of phosphorylation profiling *in vitro* using Phos-tag SDS-PAGE is highly significant. Such an analysis of phosphorylation dynamics in a two-component system had not been previously possible, because phosphoryl groups on His and Asp residues are chemically unstable and, consequently, methods such as Western blotting with antibodies for site-specific phosphorylated species or quantitative analysis of peptides with phosphorylated His and Asp residues using mass spectrometry have proved challenging. A significant enhancement of phosphorylation in the HK domain as a result of Ala substitution of the Asp residue in the receiver domain was also observed in *in vivo* phosphorylation assays of EvgS and EvgS1. The major difference between these results and those obtained from the *in vitro* assay is that the D1009A mutants were not phosphorylated at a level comparable to that observed in the *in vitro* assay because EvgS, being a membrane protein, is not able to move freely, as is the case in solution. In normal bacterial cells where EvgS is not overexpressed, the density of EvgS distribution inside the membrane is even lower, and it is therefore unlikely that ATP is wasted through signal transduction. In such a case, the precise regulation of the volume of the signal in the receiver domain would be highly significant.

It is not the aim of this report to argue which of the two-component systems, one employing a typical and simple His-Asp phosphorelay and the other employing a more complex His-Asp-His-Asp phosphorelay, is the more sophisticated. We believe that the findings of this study offer significant insights into the discussion regarding the significance of the phosphoryl-transfer reaction regulated by the more complex His-Asp-His-Asp phosphorelay.

## Supporting Information

S1 Fig Analysis of the phosphate stoichiometry of the three upshifted bands of EvgS in the autophosphorylation reaction. The phosphate incorporation ratios (^32^P signal intensity to the density of gel staining of the three electrophoresis bands of the phosphorylated EvgS in the reaction time of 30 min) were determined densitometrically. The obtained ratios (area 1'/area 1, and area 2'/area 2, and area 3'/area 3) are almost equal, indicating that the each phosphorylated EvgS form have a phosphoryl group per molecule of protein.
(TIF)

S2 Fig Autoradiography analyses of EvgS autophosphorylation, the EvgS/EvgA phosphorelay, and EvgA autophosphorylation by using Phos-tag SDS-PAGE. Each reaction was performed in the presence of 370 kBq of [γ-^32^P]-ATP at 25°C for 0–30 min. Reaction products were analyzed by Phos-tag SDS-PAGE [7% (w/v) polyacrylamide and 25 μM Mn^2+^–Phos-tag], and the phosphorylated protein bands were detected by autoradiography. The radioactivity signal of the band derived from the form phosphorylated at the H721 residue in the D1009A mutant (H721–P) was much stronger than that in the wild-type EvgS produced by the autophosphorylation and EvgS/EvgA phosphorelay.
(TIF)

S3 Fig Profiling of autophosphorylation reactions of wild-type EvgS (A) and the two mutants D1009A (B) and H1137A (C) in an ATP- and dose-dependent manner by using Phos-tag SDS-PAGE [7% (w/v) polyacrylamide and 25 μM Mn^2+^–Phos-tag]. These electrophoresis images were used in densitometric analyses to obtain the ratio values shown in Fig 5A, 5C and 5D in the main text.
(TIF)
