# Correction: Functional Characterization of the Receiver Domain for Phosphorelay Control in Hybrid Sensor Kinases

**DOI:** 10.1371/journal.pone.0136022

**Published:** 2015-08-14

**Authors:** The PLOS ONE Staff

There is an error in the last sentence of the Abstract. The publisher apologizes for the error. The correct sentence is: Furthermore, our *in vivo* assays confirmed the existence of a similar hyperphosphorylation reaction in the HK domain of the EvgS mutant in which the Asp residue was replaced with Ala, confirming the validity of the control mechanism proposed from profiling of phosphorylation *in vitro*.
