# Supplementary figures and images for: Functional Characterization of the Receiver Domain for Phosphorelay Control in Hybrid Sensor Kinases
Source: PLoS One. 2015 Jul 7;10(7):e0132598. doi: 10.1371/journal.pone.0132598 (PMC4494823; doi:10.1371/journal.pone.0132598)

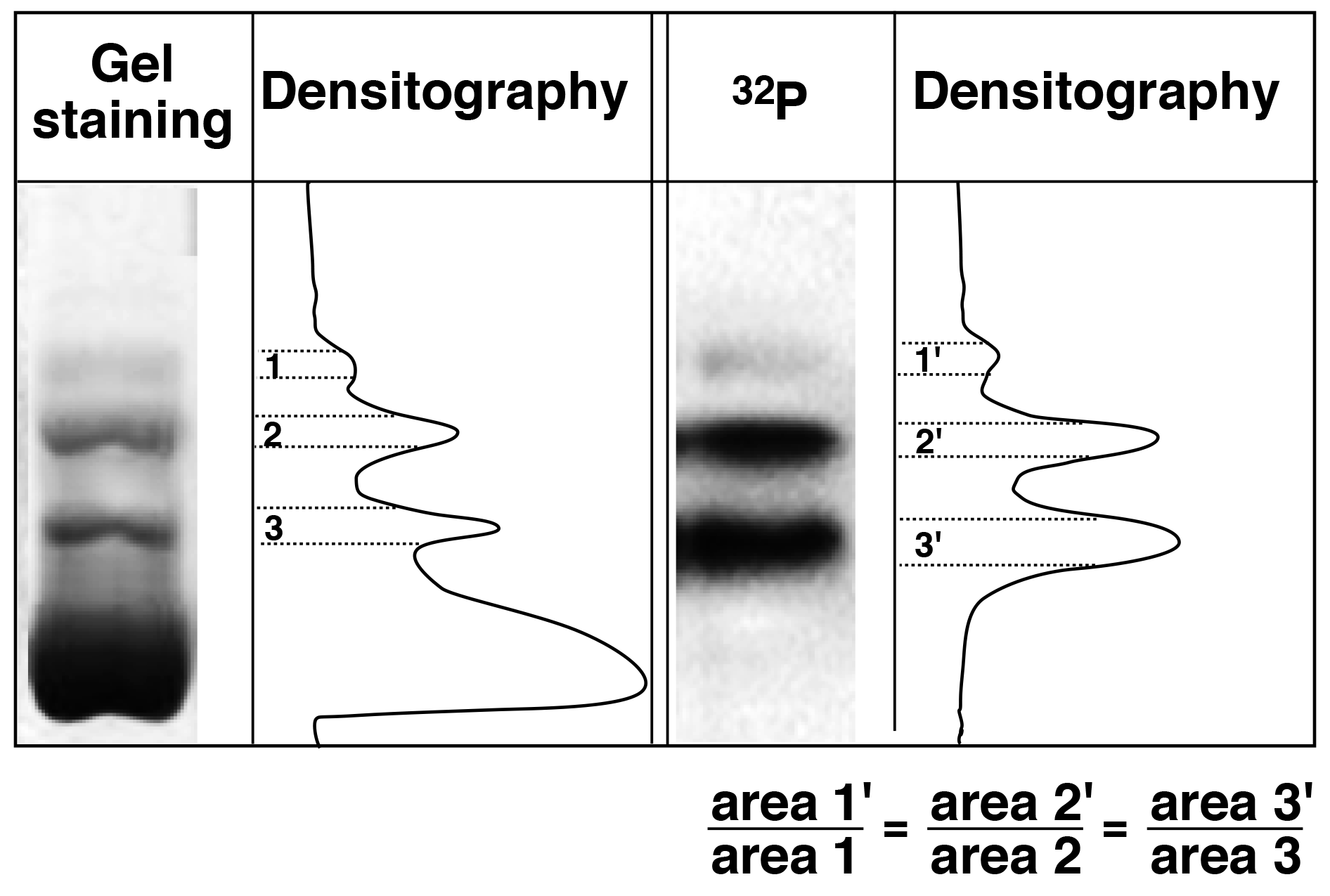

Supplement: S1 Fig — The phosphate incorporation ratios (32P signal intensity to the density of gel staining of the three electrophoresis bands of the phosphorylated EvgS in the reaction time of 30 min) were determined densitometrically. The obtained ratios (area 1'/area 1, and area 2'/area 2, and area 3'/area 3) are almost equal, indicating that the each phosphorylated EvgS form have a phosphoryl group per molecule of protein. (TIF) [file pone.0132598.s001.tif]

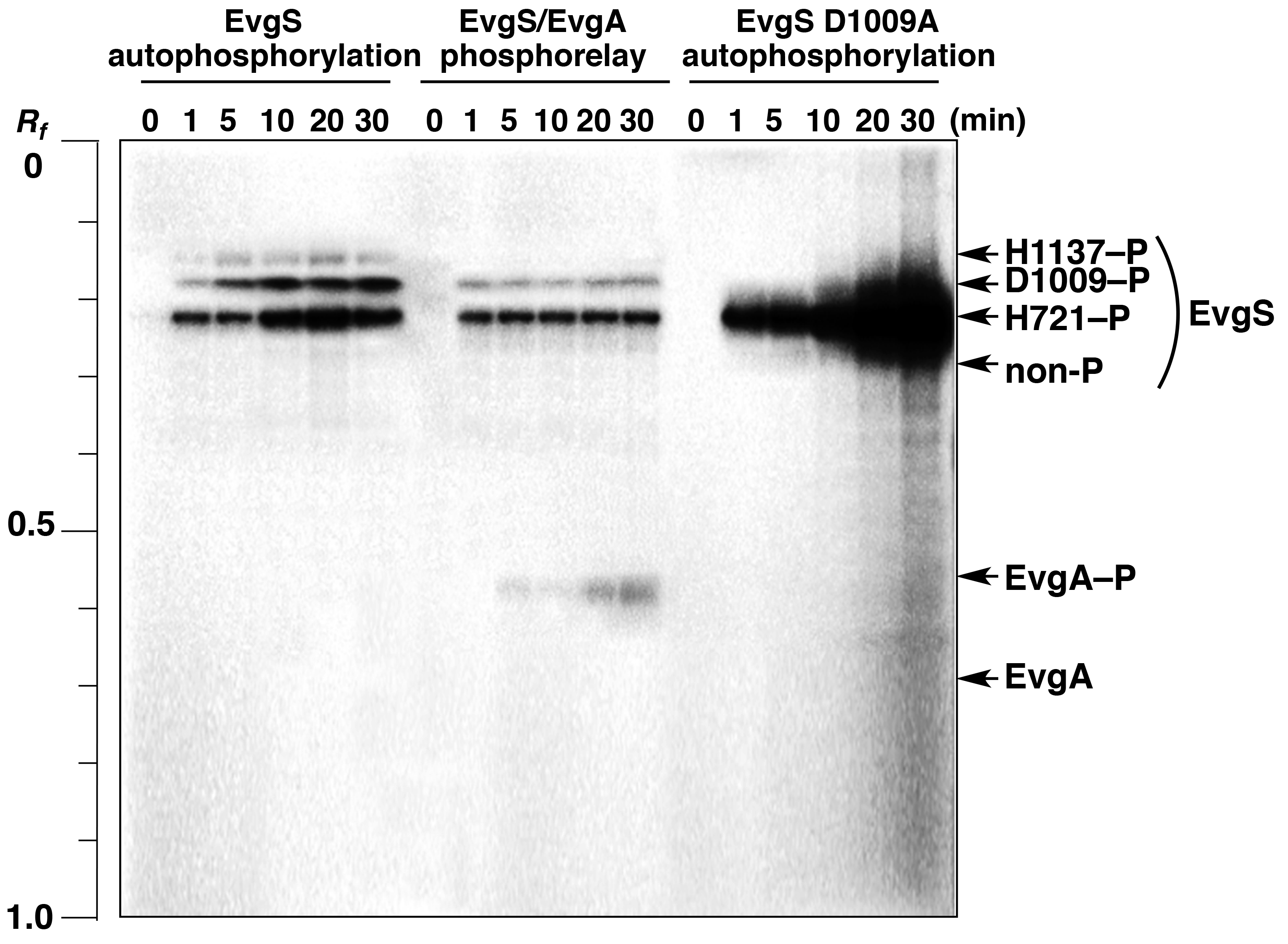

Supplement: S2 Fig — Each reaction was performed in the presence of 370 kBq of [γ-32P]-ATP at 25°C for 0–30 min. Reaction products were analyzed by Phos-tag SDS-PAGE [7% (w/v) polyacrylamide and 25 μM Mn2+–Phos-tag], and the phosphorylated protein bands were detected by autoradiography. The radioactivity signal of the band derived from the form phosphorylated at the H721 residue in the D1009A mutant (H721–P) was much stronger than that in the wild-type EvgS produced by the autophosphorylation and EvgS/EvgA phosphorelay. (TIF) [file pone.0132598.s002.tif]

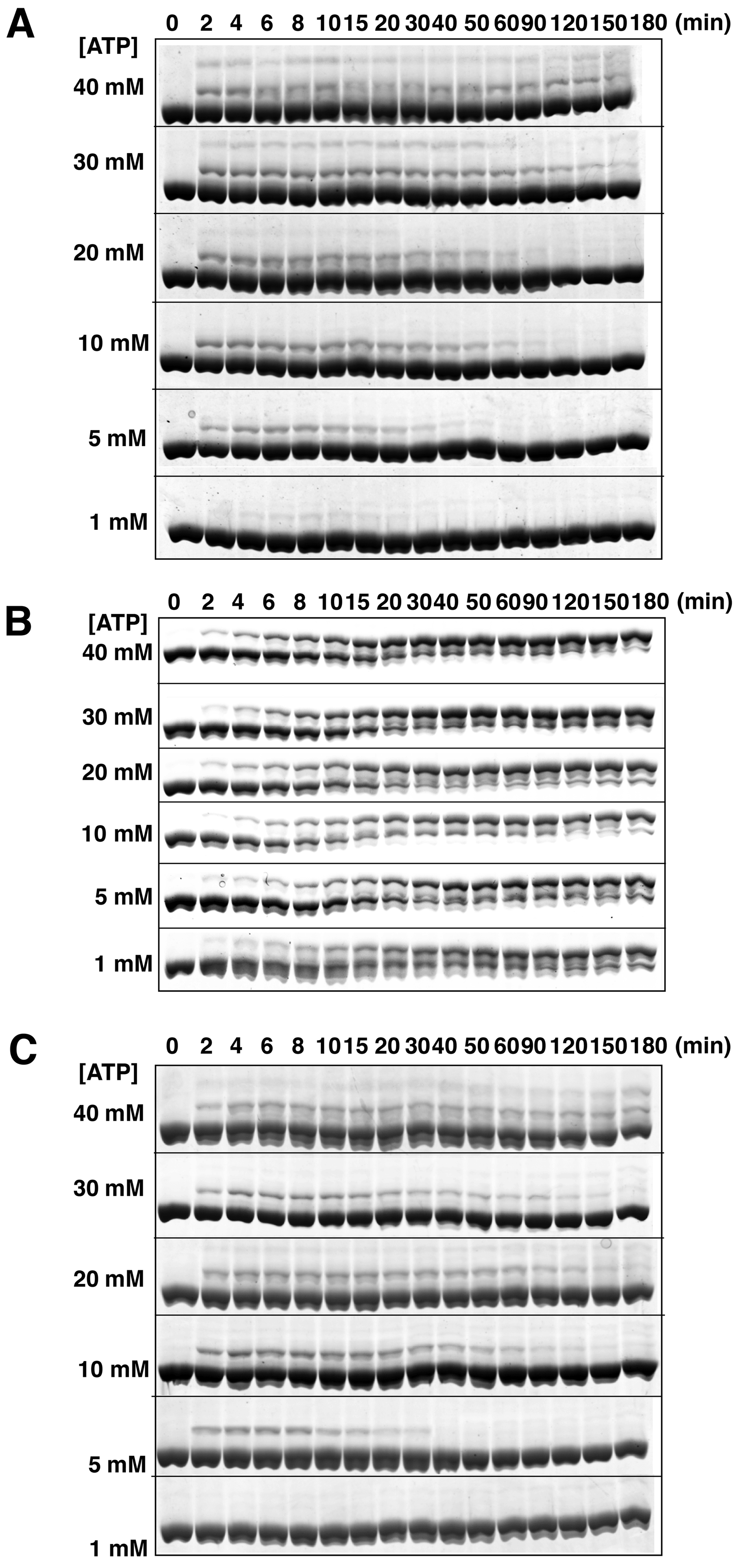

Supplement: S3 Fig — These electrophoresis images were used in densitometric analyses to obtain the ratio values shown in Fig 5A, 5C and 5D in the main text. (TIF) [file pone.0132598.s003.tif]
